# ‘Struggling to be a defender of health’ –a qualitative study on the pharmacists’ perceptions of their role in antibiotic consumption and antibiotic resistance in Romania

**DOI:** 10.1186/s40545-016-0061-y

**Published:** 2016-03-31

**Authors:** I. Ghiga, C. Stålsby Lundborg

**Affiliations:** Faculty of Medicine, Lund University, Malmo, Sweden; Department of Public Health Sciences, Karolinska Institutet, Stockholm, 171 77 Sweden

## Abstract

**Background:**

Antibiotic resistance is a serious global public health problem directly correlated to high antibiotic consumption. Romania is one of the European countries with the highest rates of antibiotic consumption, non-prescription antibiotics use and resistance of several pathogens to antibiotics. Pharmacists are an important stakeholder in respect to antibiotic management and context specific research on this topic is needed.

The aim of the research is to increase the understanding of how community pharmacists in Romania perceive their roles in respect to antibiotic consumption and antibiotic resistance.

Semi-structured interviews with 18 pharmacists were conducted to explore the perceptions and attitudes of pharmacists towards their roles on antibiotics consumption and antibiotic resistance. Manifest and latent qualitative content analysis was used to analyse interview transcripts.

**Results:**

Three sub-themes emerged from the analysis. ‘*Maintaining equilibrium between ethics, law and economy*’ expresses how pharmacists often feel when trying to fulfil their duties considering all the dimensions of the pharmacist profession.‘ *Antibiotic resistance problem rooted in a low social capital environment*’ reflects the pharmacists’ perceptions of the deep causes of antibiotic resistance and the underlying problems that perpetuate the status quo and impact their role in relation to this problem. Wanting to fulfil their educational role illustrates how the pharmacists feel they could best contribute to improving the present situation. The overarching theme ‘*Undervalued medicines’ professionals struggling with agency related and structural barriers to meet their deontological duties’*- meaning the ethical responsibilities that come with the pharmacy practice, reflects that the pharmacists see their roles as being challenged by several barriers.

**Conclusions:**

A health system and societal context perspective is helpful in order to understand the pharmacists’ roles in respect to antibiotic consumption and antibiotic resistance. Health promotion interventions and policy revisions should take into account concepts of structure and agency. These could highlight barriers that pharmacists encounter in their activities related to antibiotics management.

## Background

Antibiotic resistance is a serious global health problem and there is wide consensus that the major cause of antibiotic resistance development is use of antibiotics [1]. The WHO Regional Office for Europe has undertaken a study investigating the role of pharmacists in respect to encouraging prudent use of antibiotics and prevention of antibiotic resistance emergence and found that pharmacists are among the best positioned health professional group to tackle antibiotic resistance [1]. The following roles have been identified: the educational role of the pharmacist in respect to patients [2, 3] as well as peers [4], their duty to ensure patient compliance [5], and advise physicians on appropriate prescription [4, 6], as well as the possibility to participate in health promotion campaigns [7]. However there are also a range of determinants that affect the pharmacists’ ability to carry out their duties correctly. From a pharmacist’s point of view, dispensing antibiotics upon request to patients that self-medicate can be influenced by them knowing the patient –leading to complacency to patient’s request as shown by research conducted in Spain [8] and Portugal [9] or it can be a consequence of insufficient knowledge from the pharmacist’s side [9, 10].

Other determinants of antibiotic dispensing without a medical prescription are external responsibility and indifference, as pharmacists could tend to transfer responsibility to other actors such as other pharmacists/medical staff, patients, the healthcare system or the use of antibiotics in animals [8].

Financial considerations also alter the pharmacists’ behavior in respect to dispensing of antibiotics [11] but they depend on several characteristics of that particular setting. For example in a study set in Bulgaria, Stoyanova et. al [12] signal the influence of economic incentives of both pharmaceutical companies and consumers on the attitudes of the pharmacists which become oriented towards gaining economic benefits rather than meeting the established social and ethical norms.When considering the socioeconomic status of the population, a study in Lebanon [13] concluded that dispensing antibiotics without medical prescription in Beirut community pharmacies is especially common in lower socioeconomic areas. Pharmaceutical industry incentives and healthcare provider competition are highlighted as ethical challenges in a study in India [14].

In the EU, the data from the European Centre for Disease Prevention and Control (ECDC) show that Romania’s consumption of antibacterials for systemic use (ATC group J01) in the community (primary care sector) expressed in defined daily dose (a statistical measure of drug consumption) per 1000 inhabitants and per day in 2014 was the second highest in Europe at 31.16, while Greece has the highest at 34.04 [15]. Romania is also among the countries with the highest rates of non-prescription antibiotic use [16] as well as among those experiencing some of the highest rates of resistance of several pathogens to antibiotics in Europe in 2014, with more than 50 % of resistance for methicillin resistant *Staphylococcus aureus* (MRSA), multidrug-resistant *Klebsiella pneumoniae* (resistant to third-generation cephalosporins, fluoroquinolones and aminoglycosides) and vancomycin resistant *Enterococcus faecium* [17].

Romania is an upper-middle income country [18] that joined the EU in 2007 after going through a transition that required many changes in Romanian society following the 1989 revolution -marking the end of a communist regime which lasted 41 years. The population of Romania is 19.93 million and it has been decreasing since 1989. The negative population growth has been caused by emigration, decrease in birth rates and a rise in mortality [19]. The GINI Index, which measures income inequality (varying between 0 – perfect equality and 100- perfect inequality) was 27.3 for Romania [18]. The country’s Human Development Index value (a summary measure for assessing long-term progress in the following dimensions of human development: a long and healthy life, access to knowledge and a decent standard of living) for 2012 is 0.786, ranking 56 out of the 187 UN-recognized countries and territories [20].

The life expectancy in Romania is 74 and the total expenditure on health as a percentage of GDP (2012) was 5.1 [21]. The health care system is a decentralized and pluralistic mandatory social health insurance system organized on two levels: national and district. At the national level the Ministry of Public Health is the government institution ensuring the stewardship role of the health system while the National Health Insurance Fund is the main financial source as the third party payer of the system. This fund is an autonomous public institution. Some population groups, such as children under 18 years, are exempt from paying a contribution to the insurance fund [19].

The country is divided into 41 districts plus the region of Bucharest (the capital of Romania). Each district has a District Public Health Authority and a District Health Insurance Fund. The district institutions should ensure the provision of health services according to the rules established at the national level [19]. Another public institution is the National Agency for Medicines and Medical Devices which is subordinated to the Ministry of Public Health. Among its activities are: the medicinal product quality control, pharmaceutical inspection activity and regulatory activity under the Ministry of Public Health. The pharmacy inspection activities have also been carried out by a department located in the Ministry of Public Health.

The College of Pharmacists is the national association where all the pharmacists need to register in order to be able to practice. It has a national structure as well as local –district level ones. Currently the College of Pharmacists has approximately 14,500 members – the number of pharmacists practicing in Romania. The College of Pharmacists’ responsibilities are within the field of accreditation, control and oversight of the pharmacist’s profession including organizing courses for continuous professional education [22]. A pharmacist diploma is gained after attending pharmacy university studies which take 5 years. A pharmacist’s assistant needs to go through a post-high school specialized training that takes 3 years. The Romanian law states that opening a new pharmacy in the urban setting is done according to a demographic criteria based on the number of inhabitants- for example in Bucharest a pharmacy for every 3,000 inhabitants. Everyone can own a pharmacy and several pharmacy chains operate throughout the country. Before the 1989 Romanian revolution there were no privately owned pharmacies. Romania’s accession to the EU meant among other things harmonization of legislation as well as free movement of professionals. This had an impact on the number of medical professionals still practicing in Romania, for example 14,400 doctors were working in Romanian hospitals in 2013 compared with 21,400 in 2011 [23].

The law in Romania prohibits antibiotic sales without a medical prescription in general but it allows it in cases of medical emergencies when reaching the doctor is considered no longer possible. The sale of antibiotics over the internet is prohibited.

With all this background we explored the perceptions of Romanian pharmacists, when it comes to the role they play in antibiotic consumption and antibiotic resistance. To the best of our knowledge this is the first qualitative study on this target group, on this subject, in Romania.

## Methods

A qualitative research design was used and individual interviews were carried out. A semi-structured interview guide was created based on concepts that emerged during the literature review and included questions in the following areas: background information, pharmacist’s impressions on the patients’ knowledge and attitudes, the pharmacist-doctor collaboration, the pharmacist’s responsibilities and potential role in mitigating antibiotic resistance and national stewardship. Maximum variation sampling was sought by choosing diverse participants to allow for existing patterns among different groups to emerge [24]. Sampling of informants began purposely by contacting people working in the field, first in Bucharest, followed by contacting representatives of the College of Pharmacists throughout the country. Recruitment was also tried by posting about the research on relevant Internet social media. Providing information on the study and enquiring about potential participants was also performed directly in pharmacies in Bucharest. In some cases pharmacists that were contacted referred to other pharmacists due to their lack of time (2 people) or because they considered that they are not working enough with the patients, being focused on the managerial side of running a pharmacy (1 person). Six more pharmacists working in chain pharmacies in Bucharest were invited to participate by one of the researchers, but they declined without offering explanations. Therefore the research currently reflects the views of pharmacists working in community-independent pharmacies with only one participant having recently worked in a chain pharmacy who was no longer employed there at the time of the interview.

**Fig. 1:**
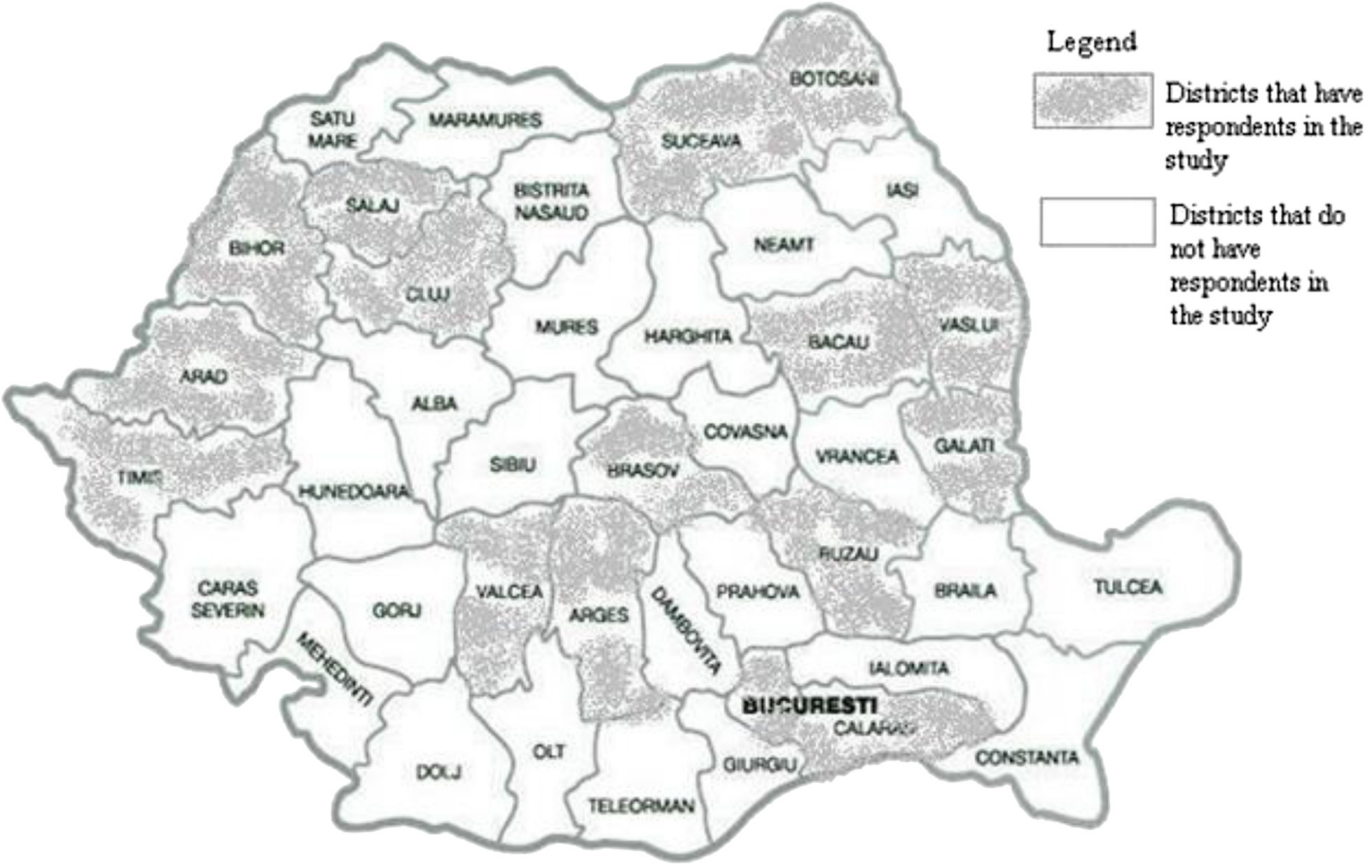
Geographical distribution of participants as per the Romanian districts

The geographical distribution of the participants is shown in Fig. 1 which shows the participants were located in 16 different districts and Bucharest. Participants were aged between 24 to 60 years and 2 were male. All participants had graduated from state-owned institutions with the following distribution of town/number of participants: Bucharest/7, Iasi/6, Cluj-Napoca/3, Targu Mures/2 and Timisoara/1.One of the interviewees worked in the rural setting while the rest worked in urban independent community pharmacies. One had experience working in chain pharmacies, no longer working there at the time of the interview, while the rest worked in independent ones. Before the actual data collection started, one pilot interview was conducted with the purpose to test the understanding of the questions in the interview guide. All interviews were conducted by the first author face-to-face (*n* = 3) or over the phone (*n* = 15) in Romanian between February and March 2015. In total 18 interviews were conducted, lasting on average 35 minutes ranging between 20 to 70 minutes. No reimbursement was offered for participation. Saturation was reached after the 16th interview after which no new significant information emerged [24] but a further 2 interviews were carried out to confirm this impression. All interviews were recorded using an electronic device, were transcribed verbatim and translated into English. Manifest and latent qualitative content analysis was used to analyse the data. This analytical approach employs codes to develop internally consistent and externally mutually exclusive categories (manifest level) that are afterwards interpreted in view of finding the underlying meaning reflected in the emergent themes (latent level) [25]. Meaning units were sections of the text that were connected to each other through content and context. Condensed meaning units were not used as the meaning units were clear enough to allow for coding without further condensation. The codes were grouped together first into sub-categories and afterwards in categories. Emerging sub-themes were constructed from the categories and finally an overarching theme was formulated which reflects the interpretation of all the text.

### Ethical considerations

The research was exempted of approval by the National Bioethics Committee of Medicine and Medical Devices in Romania and the Romanian National Bioethics Committee – UNESCO. An informed consent was signed by all the participants prior to their interview.

## Results

Three sub-themes and one overarching theme (described below) emerged from the analysis. In Table 1 categories, sub-categories, sub-themes and the overarching theme are presented.

**Table 1:** Overview of emerging categories, sub-categories, sub-themes and overarching theme

Manifest meaning	Latent meaning	
Categories (in bold) and sub-categories (plain text)	S*ub-themes*	*Overarching theme*
**Health system barriers that the patients face in accessing care**	*Maintaining equilibrium between ethics, law and economy*	***Undervalued medicines’ professionals struggling with agency related and structural barriers to meet their deontological duties***
Access to medical professionals implies various extra costs		
Financial barriers to take antibiotics correctly		
Social situations that affect vulnerable populations		
Prevention is not a focus of the system and is expensive		
**The patient’s health comes first**		
**Facing negative incentives that determine a pharmacist to push the limits of law**		
**Distrust in state institutions**	*Antibiotic resistance problem rooted in a low social capital environment*	
A flawed law that is often bent or broken		
Ministry of Public Health –a challenged institution		
A strained relationship between the state and the pharmacists		
Unreliable statistics		
Pharmacists –theoretical roles that are not translated into practice		
**Suffering from an image deficit at all levels including among themselves**		
The pharmacist perceived as a salesperson		
The pharmacist assistant – sometimes used to the detriment of the medical act		
Pharmacists university education is questionable		
Pharmacists continuous education rarely targeting antibiotics and mostly focusing on the commercial aspects of the profession		
**High rates of antibiotic consumption and antibiotic resistance (ABR) a collective fault for a neglected but serious issue**		
ABR a low-visibility problem		
High antibiotic consumption a shared blame		
**Working in silos**		
Limited or degraded institutional collaboration with the doctors		
Distrust in the doctors resources to carry out a good quality medical act		
**Education is needed**	*Wanting to fulfil their educational role*	
Patients have a low level of health literacy		
Patients having incorrect conduct in pharmacies when it comes to antibiotics		
Questionable sources of getting medical information		
Education of the patient- a sustainable solution		
**Pharmacists have the potential to educate**		
Pharmacy an accessible and welcoming environment		
Pharmacists used to fostering their diplomatic abilities		
Pharmacist a voice in the community		

**Sub-theme 1: Maintaining equilibrium between ethics, law and economy** expresses how the pharmacists often feel when trying to fulfil their duties considering all the dimensions of the pharmacist profession. The sub-theme is based on the following categories: (1) Health system barriers that the patients face in accessing care, (2) The patient’s health comes first and (3) Facing negative incentives that determine a pharmacist to push the limits of law.

***Health system barriers that the patients face in accessing care*** captures how the pharmacists perceive the patients’ situations when trying to treat their illnesses. Pharmacists are aware that *access to medical professionals implies various extra costs* which in turn dictate a certain behavior from the patient and the pharmacist.

*The majority are from the rural area, and don’t have access to a doctor or they don’t have a pharmacy in the country side, in their village […] Accessibility to the family doctor is truly hard, very difficult.[P9]*

Furthermore pharmacists expressed how they see also *financial barriers to taking antibiotics correctly.* This sub-category differentiates itself from the first as it expresses the pharmacists’ impressions on the patient’s predicament in sustaining a correct medical treatment. Another sub-category that was formed was a result of perceptions on the *social situations that affect vulnerable populations*. Pharmacists’ attitudes towards these population groups highlight the ethical dilemmas they face when patients ask for antibiotics without a medical prescription. The pharmacists describe the situation of uninsured people resulting from unemployment, a situation often faced by the Roma minority, or emigration. Pharmacists described a situation where Romanians go abroad to work, usually in low paying jobs with limited benefits, either in agriculture or construction. Before they leave, or when they come home for their annual leave to visit, they try to buy medicine in Romania to have them, if they need them, in their new country of residence, as they are often not covered by insurance there either.

*There are some patients that go abroad you know. There, they really don’t have access to the doctor. These patients ask for antibiotics, to have it there for the worst. At least in XXX, there are a lot that are away, so there is also this situation.[P9]*

Noticing these health system barriers that patients face, the pharmacists consider that ***the patient’s health comes first*** and therefore the professional behavior needs to be aligned to these considerations.

*In particular most of the patients that solicit medicines without prescription are the dentists’ patients and this is because I am speaking for XXX where the problem is that there is no on-call service for dental problems […] But you can’t go home in peace knowing that that person has a night in front of him, because even if they go to the emergency room, there, they are told that the case doesn’t belong to them [the hospital], so they can’t help him.[P14]*

The participants also expressed their predicament when ***facing negative incentives that determine a pharmacist to push the limits of law.*** This supports the dichotomous perspective on the pharmacist profession: a medical formation operating in a business environment. The interviewees talked about the impact on the pharmacist’s behavior of the distributors’ discounts for antibiotics as well as the unhealthy competition triggered by the numerous pharmacies*.*

*…in Romania these things, they are … borderline. Because if you don’t do something, you don’t make it so, you need to maintain a sort of morality and at the same time keep your business …it’s sort of difficult. [P3]*

**Sub-theme 2: Antibiotic resistance problem rooted in a low social capital environment** reflects the pharmacists’ perceptions of the underlying causes of antibiotic resistance and impact their role in respect to this problem. The following categories formed this sub-theme: (1) distrust in state institutions, (2) suffering from an image deficit at all levels including among themselves, (3) high rates of antibiotic consumption and antibiotic resistance as a collective fault for a neglected but serious issue and (4) working in silos.

***Distrust in state institutions*** signals low vertical social capital. The pharmacists consider the current legislation that allows the dispensing of antibiotics only with prescription -except for emergency situations, as *a flawed law that is often bent or broken*. The participants expressed feelings of unfairness as it would allow for unjust power asymmetries and unfair repercussions.

The pharmacists revealed distrust towards the state’s capacity to carry out their control roles. Under-staffing of these institutions, suspicion of corruption and lack of understanding of the situation on the ground were mentioned by the participants forming an image of *the Ministry of Public Health as a challenged institution*.

*so I don’t see, I don’t know how to say it… the infrastructure, they don’t have personnel for these inspections, they don’t have people that can go and check what’s really happening,..[P6]*

The interviewees perceived *a strained relationship between the state and the pharmacists* and often felt counted out from decision making.

Some pharmacists suspect *unreliable statistics*, as the method of gathering data is perceived unreliable due to unlikely admission of acts of transgression by the pharmacists who provide the data.

*I’m not interested in this, I mean the magnitude of the phenomenon cannot be measured, because no pharmacy will admit that they give without prescription as long as it’s outside the law and you can’t have a proper statistics … [P16]*

The sub-category *pharmacists –theoretical roles that are not translated into practice* illustrates how pharmacists view the theoretical possibilities for the profession and the realities of every-day practice. The participants have highlighted their dissatisfaction with the non-applicability of the role of clinical pharmacy in practice, signaling a lack of trust in the existing structures’ way of operating as well as the professional networks.

*I wish to see the day when the pharmacist will be the one who is asked in the hospital what antibiotic should be used, not which one is the cheapest one, who owns the license… [P14]*

Thinking about the experiences of dispensing antibiotics, the interviewees reflected on the existing perceptions on the profession indicating that they are ***suffering from an image deficit at all levels including among themselves.*** Every participant mentioned they felt that in some cases *the pharmacist is perceived as a salesperson*.

*In general at this hour, the patients see the pharmacist as a medicine salesperson because this is what the profession has been reduced to. [P18]*

The *pharmacist assistant – sometimes used to the detriment of the medical act* also contributed to the pharmacist’s image deficit. Participants revealed situations when the pharmacist assistant is dispensing antibiotics without prescription and is less likely to consider the associated risks in the face of pressure from the owner of the pharmacy.

The respondents’ attitudes towards their peers’ education illustrated that some view *pharmacists’ university education as questionable*. The newly established institutions are perceived as having less educational rigor. This points towards an intra-professional image deficit and a weak horizontal social capital.

In line with the intra-professional image deficit the pharmacists also expressed that *pharmacists’ continuing education rarely targets antibiotics and mostly focuses on the commercial aspects of the profession*. This also fuels the image deficit as it degrades the role of a pharmacist and the trust pharmacists place among their peers and educators.

*But it would be very good and highly recommended for these courses, that are taken to get professional points, to be on antibiotic themes, because the great majority now are about pharmacy marketing, how to associate various products –OTCs, para-pharmaceuticals, etc. Meaning a lot of companies and producers, even generics, are looking to make courses pertaining to marketing and sales…[P8]*

***The high rates of antibiotic consumption and antibiotic resistance - a collective fault for a neglected but serious issue*** signals the perceptions of pharmacists in respect to the status quo of these problems. *Antibiotic resistance, a low-visibility problem,* was seen by every participant as a serious one that is not tackled sufficiently. Pharmacists, doctors, the uneducated public that not only consumes but also may dispose of unused antibiotics in an inappropriate manner, consumption for animal husbandry, state structures that should exercise control, are points that have mentioned and formed the idea that *high antibiotic consumption is a shared blame*.

*It’s hard to answer, but some maybe think we give out too easily the medicines, but they are also responsible to the same extent because they prescribe in the same way. There are a lot of prescriptions and now with these colds they come still with antibiotics. [P4]*

When thinking about the collaboration with the doctors, the interviewees highlighted that currently professionals are ***working in silos*** and signaled a *limited or degraded institutional collaboration with the doctors*. The institutional norms of reciprocity are also questioned, as participants recalled cases when a legitimate professional concern met an arrogant, dismissive attitude from the doctors.

Furthermore the collaboration is strained by pharmacists’ *distrust in the doctors’ resources to carry out a good quality medical act*. This is a result of knowing the limited resources the doctors have in respect to performing bacterial culture and antibiotic susceptibility testing and the brief time dedicated to conducting a detailed anamnesis. The participants also perceived that patients sometimes have a diminished respect for the doctors as they are being recommended the same antibiotics each time. All these suggest a breakdown in the traditional patient- medical professional relationship, built on trust.

**Sub-theme 3 Wanting to fulfil their educational role** emerged as a concept that illustrates how the pharmacists feel they could best contribute to improving the present situation. The interviewees expressed repeatedly their concerns in relation to the (1) need for education and the (2) pharmacists potential to educate.

Often participants signaled that *patients have an incorrect conduct in pharmacies when it comes to antibiotics*. A combination of factors can lead to the patient’s inappropriate reactions when being turned down. However lack of education in general and in respect to the pharmacist’s role was always mentioned as the underlying cause in such situations.

Participants also pointed towards *questionable sources of getting medical information* by both professionals and patients. The family remains the main source of shaping sanitary behavior. Many participants suggested *patient education* in the form of formal school hygiene education programmes *as a sustainable solution* to the information deficit.

*If you made a patient understand this thing, you made another 10 understand, because he is young, in his turn he will have children, his children will learn this, the wife and so on… This education is being transmitted from generation to generation and I consider that we must start doing this and do our job! [P6]*

The ***pharmacists’ potential to educate*** was mentioned not only as a duty but also as facilitated by the pharmacy itself as it is perceived as *an accessible and welcoming environment*. The interviewees suggested that the less pronounced power asymmetry between the patient and the pharmacist, in contrast with the patient-doctor one, is a result of the patient paying at the pharmacy, therefore feeling more relaxed and confident in having a discussion with the professional.

*Pharmacists* also identified *as being a voice in the community*. They expressed their satisfaction with being appreciated and thanked for their recommendations. Often an air of nostalgia was perceived during the interviews with the older pharmacists, as they expressed how the pharmacist’s standing in their communities was in the past and how, now, it has diminished.

***The overarching theme ‘Undervalued medicines’ professionals are struggling with agency related and structural barriers to meet their deontological duties’*** speaks to all the identified sub-themes. Maintaining equilibrium between ethics, law and economy first pointed to the struggle of meeting the deontological duties- meaning the ethical responsibilities that come with the pharmacy practice. The need to follow the law, considering the health system structures is a challenge when thinking of moral agency. The sub-theme hints of the question of value as medicine professionals are being forced to respond to market incentives. The antibiotic resistance problem rooted in a low social capital environment sub-theme revealed other layers of complexity of the antibiotic consumption and antibiotic resistance phenomenon for pharmacists. The structural dimensions of vertical trust- trust in authorities, were accompanied by the horizontal social capital issues – trust between individuals and networks. The aspect of wanting to fulfil their educational role also captures the issues of value of the pharmacist’s education and ability to educate, the agency discussion in respect to the capacity of the pharmacist to act and the structural component of the how education is being provided.

## Discussion

The overarching theme suggests that the pharmacists see themselves as *‘Undervalued medicines’ professionals struggling with agency related and structural barriers to meet their deontological duties’* when it comes to antibiotic dispensing and antibiotic resistance.

The first sub-theme interprets the ethical dilemmas pharmacists encounter in trying to fulfil their professional role. This research brings forth the challenges pharmacists face when they see how the health system impacts the patient’s ability to seek care. Considering antibiotic resistance from a health system perspective implies understanding the interdependencies within and between systems [26]. The negative impact of market incentives like discounts, have been found also in previous research [12, 14, 27] where they are correlated with an increase in the sale of antibiotics without prescription and ethical issues. Farah et al. [13] found that in Beirut, Lebanon antibiotics without medical prescription were dispensed chiefly in lower socio-economic areas. Our research found that the pharmacists perceive that the patients from the lower socio-economic areas are less likely to take antibiotics correctly, invest in preventive actions and in some cases have a restricted access to the doctor. Perceptions of patients not completing the entire course of antibiotic treatment due to the high expense involved converge with previous research [28–30]. Therefore it is reinforced that the patient’s financial situation plays a great part in respect to antibiotic consumption. As interpreted by our research this impacts the pharmacist’s role as combined with structural barriers represented by the law, leads to ethical dilemmas.

The second sub-theme explores how pharmacists perceive the impact of elements that form social capital on their role as professionals in handling antibiotics and preventing antibiotic resistance. Starting from the considerations on the conceptual vagueness of social capital, Rostila [31] proposes the following definition: “*the social resources that evolve in accessible social networks or social structures characterized by mutual trust”*. This places social networks, social trust and social resources as components of social capital. Social trust influences health in two ways. Social capital can have a *compositional effect* on health as trust may influence directly individual health, but also a *contextual effect* as social trust impacts the political and social environment, the welfare regime and therefore indirectly health. When discussing the type of social capital the literature offers different classifications. Thinking about the structural dimension, the concepts of *bonding, bridging and linking capital* are employed [31]. Bonding capital refers to relations and trust that take place within the immediate groups of affiliation, while the bridging capital concerns access to a wider array of resources and happens between individuals who know that they differ in terms of socio-demographic characteristics. Linking capital is dealing with the relationships between individuals that imply a formal or institutionalized power of authority in society.

Research indicates that post-socialist, Eastern European states, such as Romania, have a low bridging and linking social capital [31, 32]. Findings from our research support this. The pharmacists distrust in state institutions indicate a low vertical level of trust and low linking capital leading to a poor contextual effect on health. The pharmacists perceive that this affects their role negatively. The question of negative impact of low vertical trust can be correlated with the findings of Collignon et al. [33]. In their research, they looked into rates of antibiotic resistance in Europe, comparing human antibiotic usage, private health care expenditure, tertiary education, the level of economic advancement (per capita GDP), and quality of governance (corruption) and found that corruption is the main socio-economic factor that explains antibiotic resistance.Previous findings pertaining to policy and regulatory issues highlight that poor capacity of implementation of legislation contributes to antibiotic resistance [28]. In our research, the image deficit and the poor collaboration with doctors are indicators of low bridging capital that among others, may lead to a poor diffusion of information and innovation. Closed networks, such as those that happen when working in silos, are at risk of perpetuating negative norms and behaviors in that network [31]. Therefore a high level of bridging capital will lead to the employment of informal social control over deviant health behaviors and will increase the feedback learning loop that could benefit all involved parties. These findings would suggest the need of a greater effort in respect to interdisciplinary collaboration and increase of bridging capital.

The perceptions in respect to the educational role the pharmacists can, and want, to play were considered also in connection to the patient’s level of medical knowledge are supported by previous research [2, 3, 5].

The overarching theme reflects the three sub-themes by employing the concepts structure and agency. Previous analysis discusses the potential of social theories that would involve these concepts in respect to promoting medication adherence [34] and suggests that pharmacy practice can improve by viewing agency and structural factors as barriers and facilitators, when it comes to patient behavior.

Germov [35] defines *structure* as “the recurring patterns of social interaction through which people are related to each other, such as social institutions and social groups” while *agency* is “the ability of people, individually and collectively, to influence their own lives and the society in which they live.” The combination of levels of intensity of either structure or agency results in various theories, as they consider whether social structures or human agency decide an individual’s behavior.

We consider that agency and structure are concepts that should be further explored in respect to the pharmacists and the stakeholders that impact their activity, as the power asymmetries that exist have a decisive impact on how the pharmacists view their role. The sub-themes highlight the agency problems linked to low bridging capital as well as the structural barriers that spring from the linking social capital. The ethical dilemmas could be a results of a combination of agency and structural factors and the same applies to the discussion on the educational aspects where a pharmacist’s agency to educate comes through chiefly. Therefore we suggest further research with a closer lens on social theories of agency and structure and concentrating on the symbolic interactions at pharmacist-patient and pharmacist-doctor level.

### Methodological considerations

Considerations on ensuring trustworthiness in this qualitative research have been taken ever since the planning stage. The criteria that were considered to ensure trustworthiness were: credibility, transferability, dependability, and confirmability [24]. Credibility refers to ensuring that the study measures what is actually intended [36]. The credibility in this study was ensured by having interviews that allowed deep, appropriate and well-saturated research data. The interviewer was familiar with the context and the culture of the participants and maximum variation sampling was sought, by having a diverse set of respondents. However, limitations in regards to sampling included the small number of participants from the rural area and those working in chain pharmacies as well as the lack of participants who gained an education from private universities. A good level of credibility was achieved by employing tactics that helped ensure honesty in informants such as the opportunity to refuse participation in the study, the researcher aiming to establish a rapport with the participants in the beginning, and emphasizing the independent status of the researcher [36]. Only one participant was familiar professionally with one of the researchers prior to the interviews. No major differences between the responses of this participant and the rest were noticed. The interpretation of the data collected was presented to one participant, who offered a positive feedback in the sensethat the emergent themes captured the respondents’ opinions. Peer debriefing also took place. The discussion section addresses the congruence with previous research. A possible limitation of the study is represented by the phone interviews. Each phone interview was recorded and the investigator tried to proceed in the exact manner as for the face-to-face ones. However, a telephone interview is considered a limitation as non-verbal communication, such as seeing facial expressions, is not possible. Therefore this could have affected the researcher’s assessment of the degree of achieved credibility.

Transferability concerns the applicability of the findings to other situations [24]. It was attempted to provide rich descriptions of the study context and of the phenomenon of antibiotic consumption and antibiotic resistance. Findings could be relevant for countries that are experiencing a similar level of social capital, governance structures, historical and cultural contexts, gross national income per capita and also have comparable regulatory frameworks in respect to antibiotics and pharmacy profession.

Dependability means that if study were to be repeated, in the same manner, it would produce similar results [24]. Dependability is considered high as in-depth methodological description to allow for repetition of the study has been done [36] as well as keeping of detailed records of the study process.

Confirmability pertains to data neutrality [24]. The interviewer took measures to limit the potential bias that could arise from her foreknowledge by being self-aware of this possibility and exhibiting deliberate naiveté. Efforts were taken for the present report, together with other documents if necessary, to ensure a step-by-step account of decisions and procedures taken in order to ensure an “audit trail”.

Considering the above mentioned strengths and limitations the study is thought to have a good degree of trustworthiness.

## Conclusions

Antibiotic resistance is a serious global public health problem and it is directly correlated to high antibiotic consumption. Romania is one of the European countries with the highest rates of antibiotic consumption, non-prescription antibiotics use and resistance of several pathogens to antibiotics.

Pharmacists are an important stakeholder in respect to antibiotic management and therefore context specific research on this topic was found adequate. The study sought to increase the understanding of how community pharmacists in Romania perceive their roles in respect to antibiotic consumption and antibiotic resistance. The research indicates that pharmacists see themselves as being undervalued health professionals, that when trying to meet their deontological duties face several agency related and structural barriers. This confirms the need to consider antibiotic resistance in a health system and societal context and design interventions that take into account concepts of structure and agency. More research is needed to understand the structural and agency rapports between all stakeholders. As antibiotic resistance has dire public health and economic consequences, these types of research and interventions would positively impact the current situation in Romania.

## Abbreviations

ECDC: European Centre for Disease Control
WHO: World Health Organization
